# Parental Feeding Practices among Brazilian School-Aged Children: Associations with Parent and Child Characteristics

**DOI:** 10.3389/fnut.2017.00006

**Published:** 2017-03-21

**Authors:** Laís Amaral Mais, Sarah Warkentin, Maria do Rosário Dias de Oliveira Latorre, Susan Carnell, José Augusto Aguiar de Carrazedo Taddei

**Affiliations:** ^1^Department of Pediatrics, Discipline of Nutrology, Federal University of São Paulo (UNIFESP), São Paulo, Brazil; ^2^Department of Epidemiology, School of Public Health, University of São Paulo (USP), São Paulo, Brazil; ^3^Johns Hopkins School of Medicine, Baltimore, MD, USA

**Keywords:** feeding practices, child nutrition, feeding behavior, parenting, parent–child relations

## Abstract

**Background:**

Children’s eating behavior, food intake, and weight status are highly influenced by parents, who shape their food environment *via* parental feeding practices. The aim of this study was to investigate associations between sociodemographic, anthropometric, and behavioral/attitudinal characteristics of parents and their 5- to 9-year-old children and a range of positive (“healthy eating guidance,” “monitoring”) and potentially negative (“restriction for weight control,” “restriction for health,” “emotion regulation/food as reward,” and “pressure”) parental feeding practices.

**Methods:**

Parents completed a questionnaire assessing parental and child characteristics. Parental feeding practices were measured using a Brazilian adaptation of the Comprehensive Feeding Practices Questionnaire. To test associations between parent and child characteristics and parental feeding practices, we ran bivariate logistic regression models with parent and child characteristics as independent variables and high (above median) scores on individual parental feeding practices as outcome variables. We then conducted multivariate logistic regression models containing all parent and child characteristics, controlling for child age and maternal education.

**Results:**

Lower parental perceived responsibility for child feeding, higher child use of screen devices, and higher child ultra-processed food intake were associated with lower scores on “healthy eating guidance” and “monitoring.” Higher parental perceived responsibility for child feeding and concern about child overweight were associated with higher scores on “restriction for weight control” and “restriction for health.” Parental perceptions of low weight and concern about child underweight, and higher perceived responsibility for child feeding, were associated with higher scores on “pressure.” Greater intake of ultra-processed foods and lower maternal age were associated with higher scores on “emotion regulation/food as reward.”

**Conclusion:**

Parental concerns and perceptions relating to child weight were predictive of potentially negative feeding practices. Higher scores on potentially negative feeding practices, and lower scores on positive parent feeding practices, were associated with poorer child diet and higher use of screen devices. Parental engagement in the feeding interaction predicted greater adoption of both potentially negative and positive feeding practices. These results support the need for policies and programs to educate parents about child feeding and help motivated parents to promote healthy lifestyles in their children.

## Introduction

Children’s eating behavior is highly influenced by parents, who are the main source of their child’s food experiences, their first nutritional educators, and act as providers, enforcers, and role models for the child (1–3). Parents determine what food is provided to the child, when and where it is eaten, and also the emotional tone of mealtimes, all of which may influence development of food preferences and eating behaviors, and child weight status (4). As children grow, parents may have less control over their child’s food intake, with teachers, peers, and media becoming bigger influences (5, 6), and children’s diet and weight status becoming more “public,” requiring an adjustment to feeding practices (7). Importantly, although parents exert influence on children, the relationship between parent and child is bidirectional, with parents also modifying their feeding practices based on their perceptions of their child’s characteristics and behaviors, and specific feeding goals parents have for their children (8).

To measure complex behaviors such as parental feeding practices, it is important to use appropriate instruments (9, 10). The Comprehensive Feeding Practices Questionnaire (CFPQ) aims to assess feeding practices of parents of 2- to 8-year olds, and is unique in its inclusion of a wider range of feeding practices compared to previous questionnaires, including positive feeding strategies, and two varieties of restriction—one motivated by health and one by weight control (11). The CFPQ was developed in the United States (US) in 2007 and has since been validated in France (12), Norway (13), Iran (14), New Zealand (15), Malaysia (16), and, most recently, Brazil (17, 18). This Portuguese version, validated in a sample of Brazilians from 5- to 9-year olds, resulted in a slightly modified, shorter version of the questionnaire, with 42 items distributed into six factors: “healthy eating guidance”—15 items (assesses how parents guide their child’s eating through encouragement, modeling, and teaching about nutrition, as well as parental involvement in feeding and the creation of healthy food environments), “monitoring”—six items (assesses how much parents keep track of unhealthy food their child eats), “restriction for weight control”—seven items (assesses the degree to which parents restrict their child’s food intake with the goal of controlling their child’s weight), “restriction for health”—five items (assesses how much parents restrict their child’s food intake with the goal of influencing their child’s health), “emotion regulation/food as reward”—five items (assesses parents’ use of food to regulate child’s emotions and/or to reward desirable behaviors), and “pressure”—four items (assesses the degree to which parents apply pressure to make their child eat more and/or a specific food) (17).

A number of previous studies have identified positive correlates of “healthy eating guidance” and “monitoring.” For example, higher “healthy eating guidance” scores have been associated with healthier dietary outcomes, such as higher intake of fruits and vegetables and lower intake of junk food (2, 19). Conversely, lesser use of this practice has been associated with higher weight in both children (20) and mothers (12). Previous studies have demonstrated a positive association between “monitoring” and higher maternal education (21), as well as a negative association with child age, consistent with growing child autonomy over diet as development progresses (22, 23). The literature is inconsistent regarding child weight status, with some studies reporting null associations (24–27) and others reporting associations between greater use of “monitoring” and higher child body mass index (BMI) (1, 28). Longitudinal analyses, which may be more helpful in establishing cause and effect, have not only found higher child BMI at baseline to be associated with increased use of “monitoring” over a 3-year period (23) but also with lower child BMI over time in children with low risk for obesity (29).

In contrast, “restriction for weight control,” which is associated with parental desire for the child to be thinner (4), is often associated with negative outcomes (30, 31). The use of this strategy may have counterproductive effects by interfering with children’s perceptions of internal cues of hunger, satiety, and appetite regulation (32). Despite the absence of an association between child food intake and the use of restrictive feeding practices in an experimental study (33), a longitudinal study by the same group of authors found that parents reporting the use of “restriction for health” with their children at age 3–5 years were more likely to have children who ate more following a negative emotions induction at age 5–7 years (34). Relationships between higher child BMI (12) and parental concern about child overweight (4) and the use of both “restriction for weight control” and “restriction for health” have also been demonstrated.

Studies investigating the use of “pressure” have largely demonstrated associations with poorer diet and lower weight. For example, several studies have reported that greater “pressure” is associated with greater intake of unhealthy foods (21), snacks (35), and energy-dense items (36). Child weight status has been negatively related to “pressure” in most studies (7, 21–31, 37–40). Parental concern about child underweight (39), and parental perceptions of their child having a small appetite (40) and being underweight (24, 30), have also been associated with greater use of “pressure.” However, Blissett and colleagues (33) and Brown and colleagues (35) found no association between “pressure” and either child food intake or weight.

The use of non-nutritive feeding practices is assessed by the CFPQ factor “emotion regulation/food as reward.” Lower scores on both practices have been associated with older child age (7, 12, 20), while higher scores have been associated with greater intake of unhealthy foods (20, 22), such as sweets (5), especially under conditions of negative emotion (33, 34). In terms of maternal characteristics, the use of “food as reward” has been found to be higher among wealthier (7) and more educated mothers (12), while younger mothers with higher BMIs reported greater use of food to regulate their child’s emotions (20).

As parental feeding practices are potentially modifiable risk factors for the development of healthy eating habits and weight status, it is important to understand both their predictors and their associations with children’s lifestyles. Notably, many of the previous studies on this topic have found inconsistent results, and many of them have considered a limited range of independent variables, using parent feeding instruments that assess negative practices only (19). The aim of the current study was therefore to investigate associations between sociodemographic, anthropometric, and behavioral/attitudinal characteristics of Brazilian parents and their 5- to 9-year-old children, and a diverse range of parent feeding practices, as assessed by the Brazilian version of the CFPQ. In accordance with the literature, we hypothesized that lower scores on positive feeding practices (“healthy eating guidance,” “monitoring”) and higher scores on potentially negative restrictive and non-nutritive feeding practices (“restriction for weight control,” “restriction for health,” and “emotion regulation/food as reward”) would be associated with higher weight in both children and mothers, higher parental concern about child overweight, and greater intake of unhealthy foods. We expected higher use of “pressure” to be associated with lower child weight, higher parental concern about child underweight and perception of child’s underweight, and greater intake of unhealthy foods.

## Materials and Methods

### Study Design

This study was a secondary data analysis of the Estudo de Práticas Alimentares, a cross-sectional study that aimed to adapt and validate the CFPQ for middle- and high-income families of preschool (18) and school-aged children (17) from Campinas and São Paulo, Brazil.

### Participants

Parents of 5- to 9-year olds for whom complete CFPQ data were obtained were included in this study. We excluded children with diseases that were related to nutrition and/or could influence parental feeding practices; siblings, in order to avoid sample unit duplication, keeping only the youngest child; children who were not in the eligible age group; children from parents who were not born in Brazil; respondents who were not the parent of the index child; parents who completed more than one questionnaire for the same child; and those with missing answers on parental feeding practice questions. To estimate sample size, a type I and a type II probability of 0.05 and 0.10, respectively, were considered. Maternal BMI equal or higher, or lower than 25.00 kg/m^2^ was used as the main independent variable for this estimation, assuming a proportion of 65 and 45% for inadequate use of parental feeding practices, among obese and non-obese mothers, respectively. The sample size estimation resulted in a minimum of 400 respondents, with a 10% addition for eventual losses, being a quarter of that obese mothers.

### Procedures

Participants were recruited from private schools in Campinas and São Paulo, Brazil. Fourteen of the 48 contacted schools accepted the invitation to participate in the study and were considered for these analyses. Survey packets with the questionnaire were left in each classroom at each participating school for distribution to eligible children, with instructions to be completed within 2 weeks by one of the parents. In one school, the questionnaires were administered and completed by parents before a parents’ and teachers’ meeting. More details on the procedures are provided elsewhere (17).

### Measures

Sociodemographic, behavioral/attitudinal, and dietary information was provided by the mother or the father of the child. The percentages of families with different amounts of minimum wage were 6.91% [five minimum wage—Brazilian minimum wage in 2014: R$724.00 (US$321.77)], 18.17% (from 6 to 10 minimum wage), 18.81% (from 11 to 15 minimum wage), 18.81% (from 16 to 20 minimum wage), and 37.30% (more than 20 minimum wage). Mean age of mothers (*n* = 654) was 38.87 ± 5.06 years, and the mean age of fathers (*n* = 638) was 41.86 ± 6.29 years. Child ultra-processed food intake was assessed by a Food Frequency Questionnaire (FFQ) specially developed for this study, and previously tested in a pilot study. Nineteen food categories were included, based on their association with obesity, their high frequency of intake in Brazilian population, and recommendations of the Dietary Guidelines for the Brazilian Population (41, 42). The mean of ultra-processed food intake (artificial juice, breakfast cereal, chips, chocolate milk, crackers/biscuits/cakes with and without stuffing, dairy desserts, fast food, ice-cream/popsicle, instant noodles, processed meat, soft drink, and sugary snacks) in the 7 days prior to questionnaire completion was calculated, and this result was dichotomized using the 25th percentile as the cutoff score. Parent and child anthropometric data were parent-reported. Parental perceived responsibility for child feeding (comprising the items “perceived responsibility for child feeding,” “perceived responsibility for child portion size,” and “perceived responsibility for adequacy of child’s intake of food groups”) and parental concern about child overweight (comprising “concern about the child eating too much,” “concern about the child having to diet to maintain a desirable weight,” and “concern about the child becoming overweight”) were derived from the Child Feeding Questionnaire (CFQ) (43). Parental concern about child underweight (comprising “concern about the child eating too little,” “concern about the child having to eat more to maintain a desirable weight,” and “concern about the child becoming underweight”) was adapted from the CFQ by changing the words “overweight” to “underweight” and “diet” to “eat more” as recommended by Musher-Eizenman and Holub (11). The response options for parental perceived responsibility for child feeding were never, seldom, half of the time, most of the time, and always. The response options for parental concern about child weight were unconcerned, a little concerned, concerned, fairly concerned, and very concerned. Parental feeding practices were assessed using the Brazilian version of the CFPQ and included “healthy eating guidance” (15 items), “monitoring” (6 items), “emotion regulation/food as reward” (5 items), “restriction for weight control” (7 items), “restriction for health” (5 items), and “pressure” (4 items) factors. The response options for CFPQ questions were never, rarely, sometimes, mostly, and always, or disagree, slightly disagree, neutral, slightly agree, and agree. The process of transcultural adaptation and validation of the questionnaire is described elsewhere (17).

### Statistical Analysis

First, we ran descriptive analyses to explore the variables in the dataset and choose appropriate cutoff scores for dichotomization based on distribution and ease of interpretation (data not shown). For analysis purposes, sociodemographic, anthropometric, and behavioral/attitudinal characteristics, and dietary intake data, were treated as independent variables, and parent feeding variables as the dependent variables. For each of the six CFPQ factors (“healthy eating guidance,” “monitoring,” “restriction for weight control,” “restriction for health,” “emotion regulation/food as reward,” and “pressure”), mean scores were calculated (possible range 1–5), then transformed into a scale ranging from 0 to 100, and dichotomized around the median. For positive parental feeding practices (“healthy eating guidance,” “monitoring”) we classified those with scores at or above the 50th centile as the reference group and those with scores lower than 50th centile as the “risk” group. For potentially negative parental feeding practices (“restriction for weight control,” “restriction for health,” “emotion regulation/food as reward,” and “pressure”), we classified those with scores lower than the 50th centile as the reference group and those with scores higher than the 50th centile as the “risk” group. In order to be included in multivariate logistic regression model, we required independent variables to have *p* ≤ 0.20 in the bivariate logistic regression models. All multivariate logistic regression models were adjusted for child sex and maternal education. Statistical significance in all models was defined as *p* ≤ 0.05 (44). All statistical analyses were performed using the Stata version 14.0 software package (45).

### Ethical Approval

This research received ethical approval from the Federal University of São Paulo (UNIFESP) ethics committee with written informed consent from all subjects in accordance with the Declaration of Helsinki. The mother or father of the children provided written informed consent for participation prior to data collection.

## Results

Of the total 1,430 survey packets distributed, 700 were considered losses due to missing data on the CFPQ, missing essential demographic and anthropometric information, or the questionnaire not being returned. After the exclusion of 71 questionnaires, there were 659 valid questionnaires (effective 46.1% response rate). More details about exclusions and losses are described elsewhere (17).

Less than a quarter of the mothers had not completed college (13.83%), less than a half had monthly incomes less than Brazilian minimum wage (43.89%), and a third were overweight/obese (33.03%). Concern about child weight was slightly higher regarding child overweight (53.72%) than underweight (45.98%). The sample was well distributed between male and female children, where 46.28% were girls. Almost 36% of the children were overweight or obese based on parent report height and weight data, and nearly half of the sample (45.07%) reported a daily screen time higher than recommended (more than 2 h/day). About 7 out of 10 children (71.02%) consumed ultra-processed food in the last 7 days prior the data collection (Table 1).

**Table 1:** **Characteristics of the sample of school-aged children enrolled in private schools of Campinas and São Paulo, 2014 (*n* = 659)**.

Variables	Reference category	Potential risk category	*n* (%) in potential risk category
**Parent characteristics**			
Maternal age	>39 years (50th centile)	≤39 years (50th centile)	361 (55.20)
Maternal education	≥college completed	<college completed	91 (13.83)
Family income (per month)	>15 Brazilian minimum wage	≤15 Brazilian minimum wage	273 (43.89)
Maternal BMI	Underweight/normal weight	Overweight/obese	215 (33.03)
Parental absence during child mealtime	None of the major meals of the week	One or more of the major meals of the week	67 (10.18)
Perceived child weight status	Very thin/slightly thin/normal	Slightly fat/fat/very fat	60 (9.12)
Perceived responsibility about child feeding	Always	Never/seldom/half of the time/most of the time	361 (54.78)
Concern about child overweight	Unconcerned/a little concerned	Concerned/fairly concerned/very concerned	354 (53.72)
Concern about child underweight	Unconcerned/a little concerned/concerned	Fairly concerned/very concerned	303 (45.98)
**Child characteristics**			
Sex	Male	Female	305 (46.28)
BMI *z*-score	Extremely underweight/underweight/normal weight	Overweight/obese/extremely obese	227 (35.86)
Screen time (per day)	≤2 h	>2 h	297(45.07)
Ultra-processed food	Not consumed	Consumed	468 (71.02)

*Brazilian minimum wage in 2014: R$724.00 (US$321.77)*.

*BMI, body mass index*.

Table 2 shows the results of bivariate logistic regression analyses investigating relationships between parent and child characteristics, and each CFPQ factor. These analyses showed that lower scores in “healthy eating guidance” were influenced by six parent–child characteristics that were eligible (*p* < 0.20) to enter the final model: four related to parent characteristics (e.g., overweight/obese mother, parental absence during child mealtime, parental perception of the child being slightly fat/fat/very fat, etc.) and two to child characteristics (e.g., greater daily screen time and ultra-processed food intake). Lower “monitoring,” on the other hand, had greater number of associations: five related to parents (e.g., less educated mothers, overweight/obese mothers, parental perception of the child being slightly fat/fat/very fat), and again the same two to child characteristics: greater screen time and ultra-processed food intake. Regarding negative feeding practices, both restrictive practices (“restriction for weight control” and “restriction for health”) showed greater influence by parent characteristics (five and six associations, compared to two associations each in child characteristics, respectively). “Pressure” was the only practice that showed an association with family income (*p* = 0.069). Besides, we found five other parent characteristics and only one child characteristic (child greater BMI *z*-score), which could have had an importance in parental use of “pressure.” The last practice “emotion regulation/food as reward” does not seem to be very influenced by parent–child characteristics, since the practice showed associations with only two parent characteristics (maternal age and perceived responsibility for child feeding), and two child characteristics (child sex and ultra-processed food intake).

**Table 2:** **Results of bivariate logistic regression analyses with parent and child characteristics as independent variables and parental feeding practices as dependent variables (*n* = 659)**.

Variables	Potential risk category	Positive feeding practices—odds of *low* scores		Negative feeding practices—odds of *high* scores			Non-nutritive use of food—odds of *high* scores
		Healthy eating guidance	Monitoring	Restriction for weight control	Restriction for health	Pressure	Emotion regulation/food as reward
		OR (*p*)					
**Parent characteristics**							
Maternal age	≤39 years	1.20 (0.243)	0.88 (0.403)	0.88 (0.405)	0.86 (0.324)	0.91 (0.550)	**1.52 (0.010)**
Maternal education	<college completed	1.15 (0.541)	1.53 (0.064)	1.35 (0.182)	1.40 (0.149)	0.88 (0.561)	1.00 (0.988)
Family income (per month)	≤15 Brazilian minimum wage	1.20 (0.250)	1.10 (0.560)	1.17 (0.331)	1.17 (0.328)	1.34 (0.069)	1.07 (0.693)
Maternal BMI	Overweight/obese	**1.95 (<0.001)**	**1.53 (0.012)**	1.37 (0.059)	1.22 (0.225)	0.88 (0.453)	1.22 (0.248)
Parental absence during child mealtime	One or more of the major meals of the week	**2.36 (0.002)**	1.23 (0.430)	1.12 (0.661)	0.98 (0.947)	1.06 (0.819)	1.28 (0.344)
Perceived child weight status	Slightly fat/fat/very fat	1.67 (0.065)	1.55 (0.115)	**10.71 (<0.001)**	**2.29 (0.003)**	**2.07 (0.013)^a^**	1.40 (0.218)
Perceived responsibility for child feeding	Never/seldom/half of the time/most of the time	**1.87 (<0.001)**	**1.57 (0.004)**	0.85 (0.316)	0.75 (0.073)	**1.53 (0.008)^b^**	**1.38 (0.045)^b^**
Concern about child overweight	Concerned/fairly concerned/very concerned	0.84 (0.274)	0.77 (0.105)	**3.78 (<0.001)**	**2.02 (<0.001)**	0.80 (0.154)	0.95 (0.748)
Concern about child underweight	Fairly concerned/very concerned	0.85 (0.309)	0.89 (0.478)	1.20 (0.242)	1.27 (0.131)	**1.60 (0.003)**	0.96 (0.804)
**Child characteristics**							
Sex	Female	0.84 (0.255)	0.93 (0.656)	**1.36 (0.048)**	0.93 (0.629)	0.93 (0.773)	**1.37 (0.047)**
BMI *z*-score	Overweight/obese/extremely obese	1.15 (0.405)	1.07 (0.686)	**3.31 (<0.001)**	**1.66 (0.002)**	1.31 (0.106)^c^	1.08 (0.632)
Screen time (per day)	>2 h	**1.95 (<0.001)**	**1.66 (0.001)**	0.90 (0.494)	1.21 (0.231)	1.02 (0.918)	1.13 (0.448)
Ultra-processed food	Consumed	**2.12 (<0.001)**	**2.21 (<0.001)**	1.02 (0.894)	**1.43 (0.039)**	1.01 (0.975)	**1.60 (0.009)**

*Brazilian minimum wage in 2014: R$724.00 (US$321.77)*.

*OR, odds ratio; BMI, body mass index*.

*Results in bold are significant (*p* ≤ 0.05)*.

*^a^Perceived child weight status as severe thinness, thinness, and normal weight*.

*^b^Perceived responsibility for child feeding: always*.

*^c^BMI/age z-score: severe thinness/thinness/normal weight*.

After controlling for child sex and maternal education, overweight/obese mothers (OR = 1.70, *p* = 0.003), as well as parents who were absent during child mealtime (OR = 2.26, *p* = 0.006) were more likely to have low scores on “healthy eating guidance.” Low parental perceived responsibility for child feeding (OR = 1.71, *p* = 0.001; OR = 1.49, *p* = 0.013), daily screen time greater than 2 h (OR = 1.71, *p* = 0.001; OR = 1.47, *p* = 0.019), and high ultra-processed food intake (OR = 1.88, *p* = 0.001; OR = 2.01, *p* < 0.001) were associated with low scores on “healthy eating guidance” and “monitoring,” respectively (Table 3).

**Table 3:** **Results of multivariate logistic regression analyses with parent and child characteristics as independent variables and parental feeding practices as dependent variables**.

Variables	Potential risk category	Positive feeding practices—odds of *low* scores		Negative feeding practices—odds of *high* scores			Non-nutritive use of food—odds of *high* scores
		Healthy eating guidance (*n* = 649)	Monitoring (*n* = 657)	Restriction for weight control (*n* = 629)	Restriction for health (*n* = 653)	Pressure (*n* = 655)	Emotion regulation/food as reward (*n* = 653)
		OR (*p*)					
**Parent characteristics**							
Maternal age	≤39 years	–	–	–	–	–	1.55 (0.008)
Maternal BMI	Overweight/obese	1.70 (0.003)	–	–	–	–	–
Parental absence during child mealtime	One or more of the major meals of the week	2.26 (0.006)	–	–	–	–	–
Perceived child weight status	Slightly fat/fat/very fat	–	–	4.60 (0.001)	1.82 (0.040)	1.88 (0.038)^a^	–
Perceived responsibility for child feeding	Never/seldom/half of the time/most of the time	1.71 (0.001)	1.49 (0.013)	–	–	1.44 (0.023)^b^	–
Concern about child overweight	Concerned/fairly concerned/very concerned	–	–	2.98 (<0.001)	1.88 (<0.001)	–	–
Concern about child underweight	Fairly concerned/very concerned	–	–	–	–	1.49 (0.014)	–
**Child characteristics**							
Sex	Female	–	–	1.43 (0.042)	–	–	–
BMI *z*-score	Overweight/obese/extremely obese	–	–	2.44 (<0.001)	–	–	–
Screen time (per day)	>2 h	1.71 (0.001)	1.47 (0.019)	–	–	–	–
Ultra-processed food	Consumed	1.88 (0.001)	2.01 (<0.001)	–	–	–	1.57 (0.014)

*Brazilian minimum wage in 2014: R$724.00 (US$321.77)*.

*OR, odds ratio; BMI, body mass index*.

*Adjusted for child sex and maternal education*.

*^a^Perceived child weight status as severe thinness, thinness, and normal weight*.

*^b^Perceived responsibility for child feeding: always*.

Perceiving the child as overweight was associated with a greater likelihood of high scores for “restriction for weight control” (*p* = 0.001) and “restriction for health” (*p* = 0.040), with odds ratios of 4.60 and 1.82, respectively. High concern about child overweight was associated with high scores on both kinds of “restriction” (OR = 2.98, *p* < 0.001 for “restriction for weight control”; OR = 1.88, *p* < 0.001 for “restriction for health”). Female gender (OR = 1.43, *p* = 0.042) and child overweight status (OR = 2.44, *p* < 0.001) were associated with high scores on “restriction for weight control.” Parent perceptions of child underweight (OR = 1.88, *p* = 0.038), high perceived responsibility for child feeding (OR = 1.44, *p* = 0.023), and concern about child underweight (OR = 1.49, *p* = 0.014) increased the likelihood of high scores on “pressure.” Low maternal age (OR = 1.55, *p* = 0.008) and high child intake of ultra-processed food (OR = 1.57, *p* = 0.014) were associated with increased likelihood of high scores on the “emotion regulation/food as reward” subscale (Table 3).

## Discussion

The aim of this study was to explore associations between the six parental feeding practices assessed by the Brazilian version of the CFPQ and various sociodemographic, anthropometric, and behavioral characteristics of parents and their 5- to 9-year-old children.

Our results revealed several associations between parent and child characteristics and lower use of positive parental feeding practices. For example, as predicted, higher maternal BMI was associated with lower use of “healthy eating guidance” (OR = 1.70, *p* = 0.003). This was also observed in a CFPQ study with American and French parents, which showed that higher maternal BMI was associated with lower use of “teaching about nutrition,” “encouraging balance and variety,” and “modeling” (OR = 0.90, *p* = 0.050), all of which practices are included in the “healthy eating guidance” factor (12). This association could reflect the promotion of obesogenic food environments among overweight mothers (25).

The development of healthy eating habits (46) and the maintenance of healthy weight status (47) among children may be aided by a structured family routine, which may be related to the use of positive feeding practices, such as “teaching about nutrition” and “modeling” (20). Consistent with these results, we found here that the absence of parents in any of the major meals per day of the week increases the likelihood of not using “healthy eating guidance” practices by more than two times. Previous research has reported a link between the presence of a parent while the child is eating and positive outcomes such as intake of fruits and vegetables, and lower risk of childhood obesity (47, 48). Parents are also responsible for setting limits for child screen time (28), a behavior that forms part of the broader family routine. In this study, we observed a negative relationship between screen time and the use of “monitoring” and “healthy eating guidance” (OR = 1.47, *p* = 0.019; OR = 1.71, *p* = 0.001, respectively). This is consistent with the results of Bost et al., who found an association between screen time and a composite factor assessing healthy feeding practices, composed of “teaching about nutrition,” “modeling,” “involvement,” “encourage balance and variety,” and “environment” (20). Television may promote excess weight by exposing the child to unhealthy food advertising, which may encourage overeating and snacking. Parents who allow greater screen time have also been found to be less concerned about their child nutrition (36).

Another finding was that parents who perceived themselves as being less responsible for their child feeding had a greater likelihood of using lower levels of “healthy eating guidance” and “monitoring,” respectively. A study of French parents of preschool-aged children also reported a positive relationship between parents’ perceived responsibility for their child’s eating and higher use of “monitoring,” “teaching about nutrition,” “modeling,” and “encouraging balance and variety” in a linear regression (4), last three of which parental feeding practices are included in the “healthy eating guidance” factor. Together, these and our findings suggest that the perception of parental responsibility for feeding is attached to beneficial outcomes for the child.

Consistent with our predictions, we also found that lower use of “healthy eating guidance” and “monitoring” increased child intake of unhealthy food by approximately two times. This is consistent with previous studies of samples from the US (20) and New Zealand (19), and a study of Latino parents in the US (2). In contrast, greater use of “modeling” was associated with decreased intake of unhealthy foods (20, 22), suggesting the use of this strategy could have a beneficial effect on children’s diet.

As expected, the use of both “restriction for weight control” and “restriction for health” were found to be associated with parental perceptions of child overweight, in the first instance even when adjusted for child BMI, with a magnitude of almost fivefold (OR = 4.60, *p* = 0.001). Consistent with this, a US study of 5-year-old girls found that mothers’ perceptions of both their daughters’ and their own overweight status significantly contributed to their level of restrictive feeding practices (30). A longitudinal study of the same cohort additionally demonstrated higher use of restrictive practices, with higher actual child weight (31). These findings may all reflect a process by which the parent desires their child to be thinner (4), stimulated by their perception of the child’s weight status, which may or may not correspond to actual child BMI (40). Notably, in the present study, child overweight was also positively associated with “restriction for weight control” (OR = 2.44, *p* < 0.001). The same result was also found in a Brazilian study of 6- to 10-year olds for “restriction” as assessed by the CFQ, although the effect size was lower (OR = 1.36, *p* < 0.001) (28). A German study developed with children from 1- to 10-year olds found a positive correlation between child’s weight and the use of “restriction” (22), while among British children 7–9 years old, a positive trend across weight groups for “restriction” was demonstrated (24).

Some level of parental concern about child weight may lead parents to reflect on and change inadequate feeding behaviors and hence be desirable. However, too much concern may lead to potentially counterproductive parental feeding practices (32, 40) such as “restriction for weight control” and “restriction for health.” Consistent with our hypothesis, in this sample, both of these practices were reported by parents who were concerned about their child being overweight, with an increased risk of almost three and almost two times for each practice, respectively. Societal and media pressures to be thin may exacerbate parental concern about child weight status (4), and this could have a greater effect on overweight mothers who may believe their children to be at risk for developing similar weight problems (30). Another study using the CFPQ found that both kinds of “restriction” were associated with parental concern about child overweight (4), while a US study including both school-aged children and adolescents found that parental concern about child weight mediated the association between child weight status and parental use of restrictive feeding practices (6).

Our finding that parents of girls used more restrictive feeding practices for weight control than parents of boys (OR = 1.43, *p* = 0.042) is consistent with previous literature from Australia (1) and Sweden (40). Parents seem to have greater concern about their daughters becoming overweight than with their sons (1, 49), likely because thinness in females is highly valued, with overweight stigmatized from early life (50). Parents may therefore feel the need to use restrictive feeding practices with their daughters in order to promote thinness (31).

Also consistent with our hypotheses, parents who perceived their child as underweight used more “pressure” (OR = 1.88, *p* = 0.038), as did parents who perceived themselves as more responsible for child feeding (OR = 1.44, *p* = 0.023), and parents who were more concerned about their child being underweight (OR = 1.49, *p* = 0.014). Parents’ concerns (39) and perceptions (24, 30, 40) about weight may be more closely related to “pressure” than actual child underweight, with parents employing this strategy when they perceive that their child is not capable of regulating his/her own intake and therefore assume responsibility for this role (30). Notably, the parental perception of child weight status may not always reflect child actual weight status, since this perception can be a reflection of many factors, such as parents’ own weight-related values and perceived difficulties with child feeding (40). Although parents have the best intentions when using controlling feeding practices such as “pressure,” this practice may lead to the child becoming more responsive to external than internal cues of hunger and satiety, which could promote the development of overweight in the future (36, 51).

As no previous reports have studied “emotion regulation/food as reward” factor as a single practice, we draw here on findings relating to “emotion regulation” and “food as reward” separately. Foods used for these non-nutritive purposes are high energy-dense and low in nutrients (12), and these practices may lead to a decreased ability to regulate internal hunger and satiety cues. Interestingly, in this study we found that mothers who reported the use of “emotion regulation/food as reward” were younger (OR = 1.55, *p* = 0.008). This result is consistent with those of a US study assessing “food as reward/emotion regulation/pressure” (20) and may be due to a lack of experience among younger mothers who could be less equipped with alternative strategies to influence their children’s behavior.

Consistent with previous findings associating higher scores on both “food as reward” (5, 22) and “emotion regulation” (20) with higher consumption of unhealthy foods, we observed a similar association in the present study (OR = 1.57, *p* = 0.014). In further support of this finding, UK studies found that the parental use of food to regulate emotions was associated with higher consumption of cookies and chocolates in the absence of hunger (33), and this effect was increased in the context of negative emotion after a 2-year follow-up (34). Together, these findings suggest that this practice may teach the child to deal with negative emotions through palatable food intake, which provides an immediate short-term remedy (33). Some evidence suggests that the use of food as reward may increase the preference for reward foods (50). However, it is also possible that children who are particularly responsive to these foods are more likely to have such foods offered to them as a reward (5, 22).

Our study had some limitations. First, the cross-sectional design of the study does not provide evidence for causality, since it is not possible to show the direction of the influence, only associations. However, longitudinal studies addressing associations between parental feeding practices and child weight status, food intake, and other variables of interest have demonstrated that these relationships may be bidirectional (6, 26, 27, 29). Despite our attempts to include as many participants in the study as possible, there were considerable losses; nevertheless, our response rate afforded us sufficient power to detect our associations of interest. Although previous studies have shown associations between parental education and income, and the use of specific feeding practices (30, 39), our study was unable to detect these differences. This was probably due to the homogeneity of the sample, in which educational level and socioeconomic status were relatively high. Comparisons with other populations should therefore be made with caution. Future research with a more diverse sample may help to highlight parental characteristics to consider when developing interventions to modify feeding practices to optimize child health and weight status and could enable targeting of specific subpopulations (12). The fact that the majority of the respondents were mothers means that the results may not apply to other caregivers; however, mothers still shoulder most of the responsibility for feeding the child at home, making our findings relatively generalizable (28, 30).

Although self-report is a practical way to assess specific information in a large-scale survey, the self-reported nature of the anthropometric, dietary, and parental and child behaviors measures is subject to social desirability and perceptual biases (40, 52). However, parents’ perceptions may directly influence their feeding practices, making them important to study (4, 22, 53). Since a validated short FFQ for school-aged children was not available for Brazil we developed our own instrument, so comparing between studies is not possible. Finally, many other potential determinants of parental feeding practices, e.g., parental food intake and body self-image were beyond the scope of this research and therefore not measured here.

Strengths of our design include the previous translation and validation of the CFPQ in a Brazilian sample of 5- to 9-year-old children, which provides confidence that the parental feeding practices assessed here were appropriate for the sample, facilitating generalization of the findings to populations with the same characteristics. The CFPQ is that it is a practical and comprehensive tool assessing a wide range of parental feeding practices, including positive practices, and making a distinction between “restriction,” motivated by weight control, and by health, which is not a feature of most parent-feeding questionnaires. The use of a large sample including 659 participants maximized our power to detect meaningful associations. We included a wide spectrum of predictors, including sociodemographic, anthropometric, behavioral/attitudinal, and dietary variables, as well as parental concerns and perceptions regarding child weight status and food intake. This is one of the first studies of parental feeding practices to be conducted in Brazil, a country undergoing nutrition transition in which child obesity rates are growing. Understanding the role of parents in child feeding in countries like Brazil is an important foundation for the development of policies and programs to promote healthy eating and weight at individual and community levels.

To conclude, in this study of parental feeding practices in Brazilian school-aged children, we observed associations between lower use of “healthy eating guidance” and “monitoring” and negative child behaviors such as unhealthy eating. Families may benefit from increasing their use of these positive feeding practices. Conversely, the non-nutritive use of food and negative feeding practices such as “emotion regulation/food as reward,” “restriction for weight control,” “restriction for health,” and “pressure” were shown to be associated with negative outcomes, such as child overweight and the consumption of ultra-processed food, suggesting that these practices should be discouraged. Parental concerns and perceptions, more than actual child weight status were significant predictors of both positive and coercive parental feeding practices. Policies and programs that promote parents’ ability to perceive their child weight status correctly, and provide them with strategies for healthy feeding practices, should therefore be merited. The parental and child characteristics highlighted in this study could also be used to develop interventions targeted at specific populations at risk of obesogenic feeding practices.

## Author Contributions

LM contributed to the study design, participated in the data gathering and entering, performed the data analysis and interpretation, and wrote the article. SW contributed to the study design, participated in the data gathering and entering, and contributed to data analysis and interpretation and writing the article. ML suggested and supervised the data analysis and interpretation and reviewed the article. SC contributed to writing the article. JT selected the study design, supervised the data gathering and data entering, and reviewed the article. All authors approved the contents of the manuscript.

## Conflict of Interest Statement

The authors declare that the research was conducted in the absence of any commercial or financial relationships that could be construed as a potential conflict of interest.

## Abbreviations

BMI, body mass index; CFPQ, Comprehensive Feeding Practices Questionnaire; CFQ, Child Feeding Questionnaire; EPA, Estudo de Práticas Alimentares; FFQ, Food Frequency Questionnaire; UNIFESP, Federal University of São Paulo; US, United States.
